# Response of Soil Properties and Microbial Communities to Agriculture: Implications for Primary Productivity and Soil Health Indicators

**DOI:** 10.3389/fpls.2016.00990

**Published:** 2016-07-12

**Authors:** Pankaj Trivedi, Manuel Delgado-Baquerizo, Ian C. Anderson, Brajesh K. Singh

**Affiliations:** ^1^Hawkesbury Institute for the Environment, Western Sydney University, Penrith South, NSWAustralia; ^2^Global Centre for Land Based Innovation, Western Sydney University, Penrith South, NSWAustralia

**Keywords:** soil health, indicators, agriculture intensification, soil bacteria

## Abstract

Agricultural intensification is placing tremendous pressure on the soil’s capacity to maintain its functions leading to large-scale ecosystem degradation and loss of productivity in the long term. Therefore, there is an urgent need to find early indicators of soil health degradation in response to agricultural management. In recent years, major advances in soil meta-genomic and spatial studies on microbial communities and community-level molecular characteristics can now be exploited as ‘biomarker’ indicators of ecosystem processes for monitoring and managing sustainable soil health under global change. However, a continental scale, cross biome approach assessing soil microbial communities and their functional potential to identify the unifying principles governing the susceptibility of soil biodiversity to land conversion is lacking. We conducted a meta-analysis from a dataset generated from 102 peer-reviewed publications as well as unpublished data to explore how properties directly linked to soil nutritional health (total C and N; C:N ratio), primary productivity (NPP) and microbial diversity and composition (relative abundance of major bacterial phyla determined by next generation sequencing techniques) are affected in response to agricultural management across the main biomes of Earth (arid, continental, temperate and tropical). In our analysis, we found strong statistical trends in the relative abundance of several bacterial phyla in agricultural (e.g., *Actinobacteria* and *Chloroflexi*) and natural (*Acidobacteria, Proteobacteria*, and *Cyanobacteria*) systems across all regions and these trends correlated well with many soil properties. However, main effects of agriculture on soil properties and productivity were biome-dependent. Our meta-analysis provides evidence on the predictable nature of the microbial community responses to vegetation type. This knowledge can be exploited in future for developing a new set of indicators for primary productivity and soil health.

## Introduction

Soil health is the capacity of a soil to function, within natural or managed ecosystem boundaries, to sustain plant productivity, maintain water and air quality, support human well-being, and provide habitats for biodiversity (Doran and Zeiss, 2000; Doran, 2002; Gugino et al., 2009). Human impacts on soil health largely emerge from the need to meet the food, fiber, and fuel demands of an ever increasing population. In the last few decades significant efforts have been made to increase agricultural productivity through increased fertilization and pesticide application, improved irrigation, soil management regimes and crops, and massive land conversions (Tilman et al., 2002). There is increasing concern, however, that agricultural intensification is placing tremendous pressure on the soil’s capacity to maintain its other functions leading to large-scale ecosystem degradation and loss of productivity in the long term (Tilman et al., 2001; Foley et al., 2005; Vitousek et al., 2009). For example, conversion of natural ecosystems to agricultural lands have incurred substantial environmental costs, including desertification, increased emissions of greenhouse gasses, decreased organic matter in soils, loss of biodiversity, and alterations to biogeochemical and hydrological cycles (Balmford et al., 2005). Modern agriculture thus faces great challenges not only in terms of ensuring global food security by increasing yields but also mitigating the environmental costs particularly in the context of a changing environment and growing competition for land, water, and energy (Chen et al., 2014). Therefore, there is an urgent need to find early indicators of soil health degradation in response to agricultural management (Grime, 1997; Cardoso et al., 2013).

Different terrestrial biomes may respond differentially to agricultural over-exploitation. For instance, arid lands, which occupy 40% of the globe and sustain 38% of the human population (Millennium Ecosystem Assessment [MEA], 2005), are very low productivity systems and contain low levels of nutrients (Reynolds et al., 2007; Feng and Fu, 2013). These ecosystems are highly vulnerable to global environmental changes and desertification (Reynolds et al., 2007; Dai, 2013) and may further suffer high reductions in nutrient availability in response to agricultural over-exploitation (Delgado-Baquerizo et al., 2013). On the other hand, highly productive agro-systems such as those from tropical regions may be highly resistance/resilience to agriculture uses, in part due to their rapid organic matter turnover and moisture/water availability (Schlesinger and Bernhardt, 2013). Limited effort has been made to understand the global trends that characterize microbial community composition in natural and agricultural systems (Crowther et al., 2014) which hinder our ability to anticipate the consequences of conversion in the different biomes on Earth.

Evaluation of soil health requires indicators of chemical, physical and biological (including microbial) components contribute to maintaining soil health. Cultivation is known to generally reduce the amount of soil organic matter thus reducing nutrient availability (Schlesinger and Bernhardt, 2013). Similarly, changes in land use are altering both microbial community structure and diversity in terrestrial ecosystems (Rodrigues et al., 2013). Since soil bacterial communities drive many different ecosystem functions (e.g., Delgado-Baquerizo et al., 2016b), and their abundance, richness, and composition are sensitive to the changes in the land use and management (Gans et al., 2005; Wall et al., 2010; Singh et al., 2014), they have been considered as early indicators of change in the quality of soil ecosystems (Kennedy and Stubbs, 2006). In some instances, changes in microbial populations or activity can precede detectable changes in soil physical and chemical properties, thereby providing an early sign of soil improvement or an early warning of soil degradation (Pankhurst et al., 1997; Nielsen et al., 2002). At local scale fluctuations in microbial diversity and community composition are correlated with reductions in soil C and nitrogen (N) (Acosta-Martinez et al., 2008, 2010; Jangid et al., 2008; Trivedi et al., 2015). On global scale, however, land use change to agriculture systems on the soil C and N contents are more idiosyncratic (Johnson and Curtis, 2001), and negligible effect of conversion has been reported on microbial biomass from several biomes (Holden and Treseder, 2013). Since microorganisms are involved in many soil processes, they may also give an integrated measure of soil health, an aspect that cannot be obtained with physical/chemical measures alone (Nielsen et al., 2002; Kibblewhite et al., 2008; Mueller et al., 2010; Sharma et al., 2011). In recent years, major advances in soil meta-genomic and spatial studies on microbial communities and community-level molecular characteristics can now be exploited as ‘biomarker’ indicators of ecosystem processes for monitoring and managing sustainable soil health under global change. However, a continental scale, cross biome approach assessing soil microbial communities and their functional potential to identify the unifying principles governing the susceptibility of soil biodiversity to land conversion is lacking.

In the face of current anthropogenic pressure on soil ecosystems, for instance owing to agricultural intensification and climate change, there is a need to better understand the effects of these factors in order to predict and mitigate the impacts of such changes (Kuramae et al., 2012). However, reliable predictions of the potential consequences of perturbations are hampered by the lack of global level baseline knowledge on soil properties and soil microorganisms. Herein we conducted a meta-analysis to explore how soil properties (pH, total C and N; C:N ratio), primary productivity (NPP) and microbial diversity and composition (relative abundance of major bacterial phyla) are affected in response to agricultural management across the main biomes of Earth (arid, continental, temperate and tropical). The aim of the meta-analysis was to identify the impact of agriculture practices on soil nutritional health and microbial communities. We also aimed to examine if the response of microbial community to agriculture is consistent across all the biomes. We collected data from 102 peer-reviewed publications as well as unpublished data to create a global dataset of soil bacterial diversity and composition evaluated with next generation sequencing techniques (mostly 454 Pyrosequencing). Our meta-analysis revealed foreseeable nature of the microbial community responses to vegetation types suggesting that the microbial indicators can be developed as tools for prediction for primary productivity and soil health.

## Materials and Methods

### Data Collection

**FIGURE 1:**
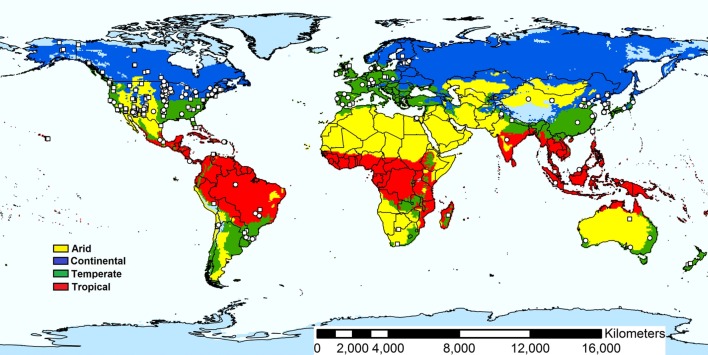
**Locations of the soil samples included in this study.** Agricultural soil samples (*n* = 165) and natural soil samples (*n* = 353) are shown as circles and squares, respectively. The sites were selected based on a meta-analysis consisting of both published and unpublished data wherein bacterial diversity and compositions is described based on next generation sequencing techniques (either 454 or Miseq) from both. From those experimental studies that manipulated environmental conditions (e.g., nutrients or climatic conditions) we only used the data from the control treatment (See Material and Methods for more details).

We collected data on soil bacterial diversity based on next generation sequencing techniques (either 454 or Miseq) from both published and unpublished data. We first conducted a search using SCOPUS^1^ (on September 2014). The following keyword combinations were used: (1) “bacterial community” AND “soil” AND “Pyrosequencing”; and (2) “bacterial community” AND “soil” AND “Illumina.” We found ~300 references. Within these references, studies were chosen for inclusion in our analyses only if they met the following criteria: (1) were carried out in the field in terrestrial ecosystems, (2) contained the spatial location where they were carried out (latitude and longitude), as well as data on soil total C and pH; (3) provided information on Shannon bacterial diversity at 97% of similarity; (4) included data on the relative abundance of soil bacterial phyla, (5) used next generation sequencing techniques (either 454 or Miseq) and (6) were located in arid, continental, temperate or tropical ecosystems (Koppen classification; Kottek et al., 2006). From those experimental studies that manipulated environmental conditions (e.g., nutrients or climatic conditions) we only used the data from the control treatment. The dataset included geographical locations covering all continents and biomes where agriculture is in practice (**Figure 1**; Data Sheet S1).

Technical information related to this study (e.g., primer sets, sequencing technology) and validation of the impact of various approaches to generate data on the conclusions is largely discussed in Delgado-Baquerizo et al. (2016a).

### Microbial Related Parameters

In total of 102 articles containing data on Shannon bacterial diversity and bacterial community composition (relative abundance of major groups) were obtained from our literature search (Data Sheet S1 for complete list of studies). We completed this database with data from the National Ecological Observatory Network (NEON; 24 soil samples) in the United States of America^2^, Canadian MetaMicroBiome Library^3^ (11 soil samples) and with unpublished data from sites in Australia (12 soil samples) and Scotland (6 soil samples) (data available from authors). From our meta-analysis we obtained a total of 518 independent soil samples (353 and 165 soil samples belong to natural and agricultural systems, respectively). Bacterial Shannon diversity and composition was available for 61 and 100% of these sites, respectively.

For all the samples available, we gathered data on the relative abundance of the following major bacteria phyla: *Proteobacteria, Acidobacteria, Actinobacteria, Verrucomicrobia, Bacteroidetes, Chloroflexi, Cyanobacteria, Firmicutes, Gemmatimonadete, Plantomycetes*.

### Soil Properties and Net Primary Productivity (NPP)

We collected information on the following soil properties: soil total C, total N, C:N ratio and pH from the studies selected for meta-analysis. Most of the studies in our meta-analysis used elemental CNH analyzer for the estimation of soil C. This method analyzes both inorganic and organic carbon hence can overestimate the amount of SOC in the samples. Data for NPP (g C m^-2^ d^-1^) was calculated from MODIS satellite imagery data as a monthly average from the 2004 to 2013 period using information with a 0.1° spatial resolution^4^.

### Ecosystem Classification

We determined the main climate classes in each of the study sites based on global maps available for the most frequently used Köppen climate classification map (Kottek et al., 2006): A (tropical), B (arid), C (temperate), and D (continental). We completed climate gaps in our dataset using local and regional database. These analyses were done with ESRI ArcGIS Desktop 10.

### Statistical Analyses

We used two-way ANOVAs to evaluate changes in NPP, soil properties and microbial community features (main bacterial phyla and Shannon diversity) among different biomes (arid, temperate, continental and tropical) and land use type (agricultural vs. natural). Biomes and land use type were included as fixed factors in these analyses. These statistical analyses were carried out using IBM SPSS 15.0 (SPSS Inc, Chicago, IL, USA). We also used Spearman correlation analyses to evaluate the relationship between microbial community features (main bacterial phyla and Shannon diversity) with soil properties and NPP.

## Results

### NPP and Soil Properties of Agricultural vs. Natural Soils in Different Regions

**FIGURE 2:**
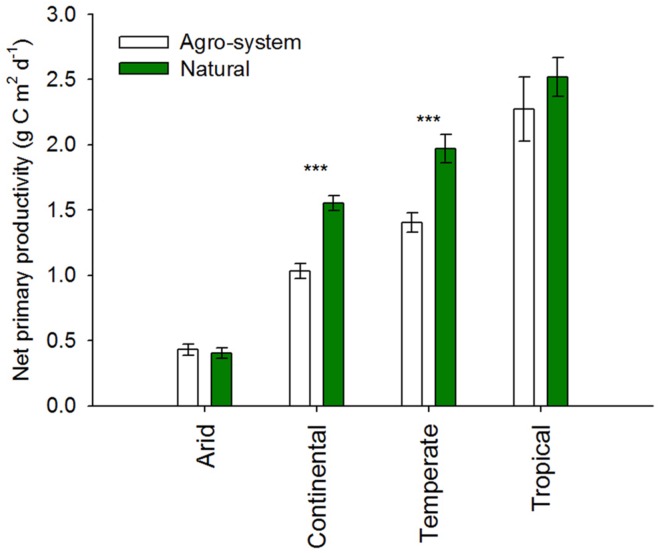
**Net primary productivity (measured as g C m^2^ d^-1^) of agricultural vs. natural systems in arid (*n* = 26 and 70), continental (*n* = 43 and 119), temperate (*n* = 82 and 128) and tropical (*n* = 14 and 36) regions.** Data for primary productivity (g C m^-2^ d^-1^) was calculated from MODIS satellite imagery data as a monthly average from the 2004–2013 period using information with a 0.1° spatial resolution (http://neo.sci.gsfc.nasa.gov/). The main climate classes are based on global maps available for the most frequently used Köppen climate classification map (Kottek et al., 2006). ***P < 0.0001.

**FIGURE 3:**
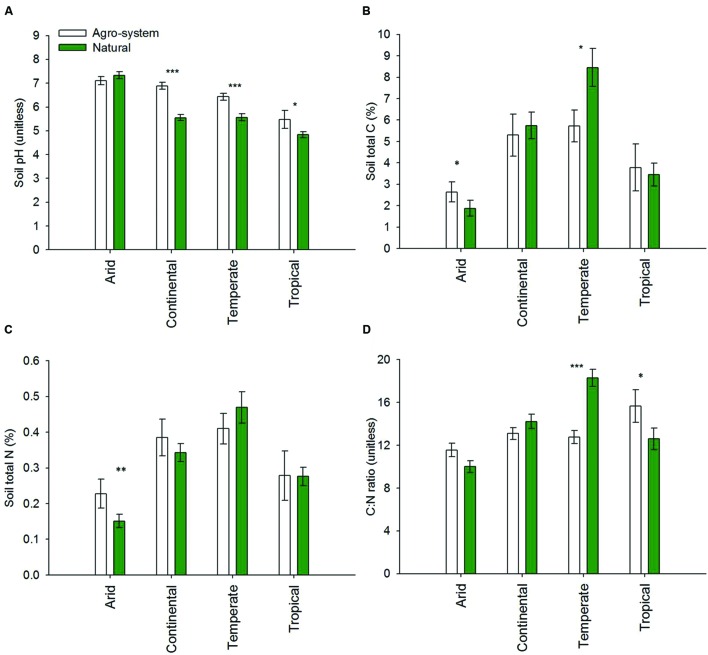
**Soil chemical properties [(A) Soil pH; (B) Soil total C (%); (C) Soil total N (%); and (D) C/N ratio] of agricultural vs. natural systems in arid (*n* = 26 and 70), continental (*n* = 43 and 119), temperate (*n* = 82 and 128) and tropical (*n* = 14 and 36) regions.** The sites were selected based on a meta-analysis consisting of both published and unpublished data wherein bacterial diversity and compositions is described based on next generation sequencing techniques (either 454 or Miseq; see Material and Methods for more details). The main climate classes are based on global maps available for the most frequently used Köppen climate classification map (Kottek et al., 2006). *P < 0.01; **P < 0.001; ***P < 0.0001.

Net primary productivity (measured as g C m^2^ d^-1^) was significantly higher in natural as compared to agro-systems from continental and temperate regions (*P* < 0.0001) (**Figure 2**). However, our meta-analysis did not reveal a significant difference between agricultural and natural ecosystems for arid and tropical regions (**Figure 2**). pH of agriculture soils from continental, temperate, and tropical regions were higher than natural soils (**Figure 3A**). The data revealed an approximate increase of 1.5, 1.0 and 0.5 units in soil pH from agriculture soils as compared to natural soils in continental (*P* < 0.0001), temperate (*P* < 0.0001) and tropical (*P* < 0.01) regions, respectively. There was no difference in the pH values associated with agricultural vs. natural soils in arid regions.

Soil % C in natural soils from temperate regions was approximately 8.0% and this was significantly higher (*P* < 0.01) than the agricultural soils (~5.5%, **Figure 3B**). The data revealed no significant difference in the % of total C in agricultural vs. natural soils from continental and tropical regions. In arid regions, our meta-analysis revealed significantly higher (*P* < 0.01) % of total C in agricultural as compared with natural soils. In arid regions the % of total C was approximately 2.5 and 1.7% for agricultural and natural soils, respectively.

Our analysis revealed variability in the soil N content when comparing agricultural vs. natural soils from different regions. In arid regions, the total N % in agricultural soils was approximately 0.22% which was significantly higher (*P* < 0.001) than natural soils (~0.15%, **Figure 3C**). We observed no significant trends in % of soil N in agricultural vs. natural soils in the other three regions. However, agricultural soils from continental regions had a higher N content compared to natural soils while the opposite trend was observed in temperate regions. The % N of agricultural and natural soils was similar in tropical regions.

As an average, arid and temperate regions showed the lowest and highest C:N ratio in this study, respectively (**Figure 3D**). The C:N ratio of agricultural soils from arid and tropical regions was higher (*p* < 0.01) when compared to natural soils from similar regions. In contrast, the C:N ratio of natural soils was significantly higher (*P* < 0.0001) than agricultural soils in temperate regions. In continental regions there was no significant difference in the C:N ratio from agricultural and natural soils.

### Microbial Data

#### Microbial Diversity in Agricultural vs. Natural Systems in Different Regions

**FIGURE 4:**
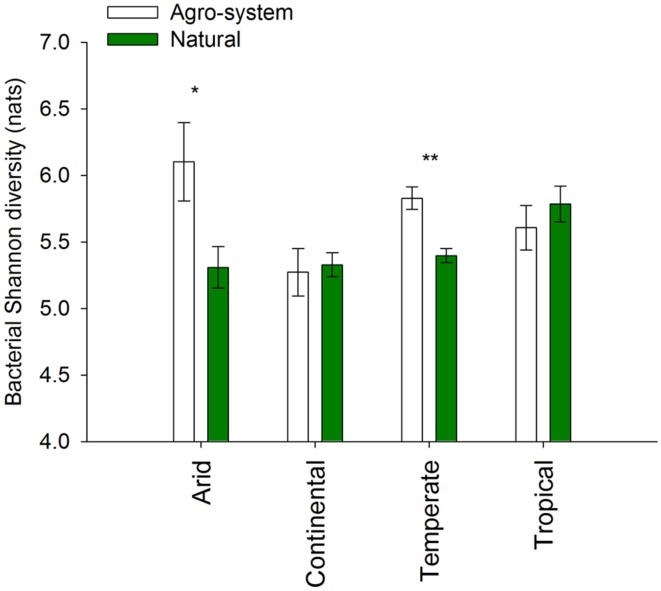
**Bacterial Shannon diversity of agricultural vs. natural systems in arid (*n* = 26 and 70), continental (*n* = 43 and 119), temperate (*n* = 82 and 128) and tropical (*n* = 14 and 36) regions.** The sites were selected based on a meta-analysis consisting of both published and unpublished data wherein bacterial diversity and compositions is described based on next generation sequencing techniques (either 454 or Miseq; see Material and Methods for more details). The main climate classes are based on global maps available for the most frequently used Köppen climate classification map (Kottek et al., 2006). *P < 0.01; **P < 0.001.

We selected the Shannon diversity index as our metric of alpha diversity because it is highly recommended and commonly used when analyzing microbial diversity (He et al., 2013), and has been shown to reduce the bias in relation to other diversity metrics, such as the number of OTUs, when comparing data from multiple sources (He et al., 2013). Our analysis revealed significantly higher microbial diversity in agricultural systems as compared to natural systems in arid (*P* < 0.01) and temperate (*P* < 0.001) regions (**Figure 4**). Microbial diversity was lower in agricultural systems in continental and tropical regions as compared with natural systems; however, the trends were not statistically significant.

#### Relative Abundance of Bacterial Phyla in Agricultural vs. Natural Soils in Different Regions

**FIGURE 5:**
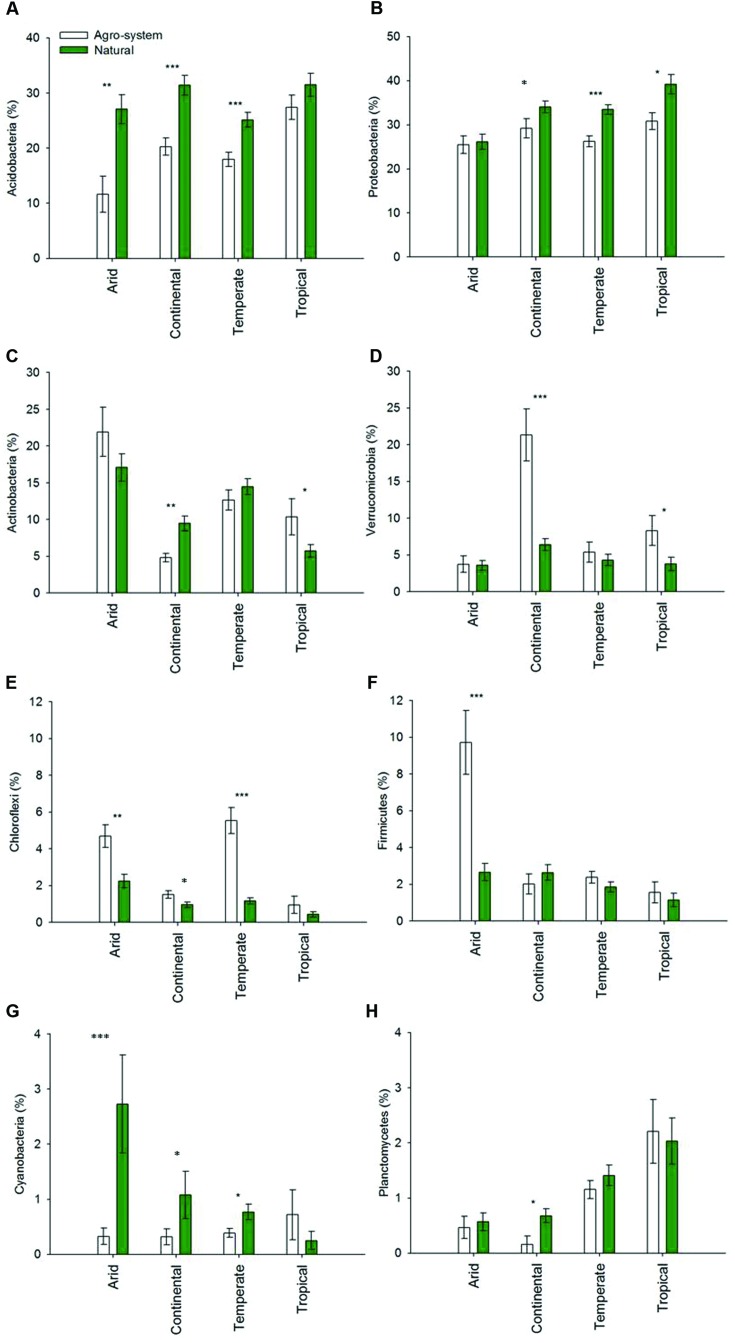
**Relative abundance of major bacterial phylum [(A) *Acidobacteria*; (B) *Proteobacteria*; (C) *Actinobacteria*; (D) *Verrucomicrobia*; (E) *Chloroflexi*; (F) *Firmicutes*; (G) *Cyanobacteria*; and (H) *Planctomycetes*] in agricultural vs. natural systems in different regions.** The sites were selected based on a meta-analysis consisting of both published and unpublished data wherein bacterial diversity and compositions is described based on next generation sequencing techniques (either 454 or Miseq; see Material and Methods for more details). The main climate classes are based on global maps available for the most frequently used Köppen climate classification map (Kottek et al., 2006). *P < 0.01; **P < 0.001; ***P < 0.0001.

The relative abundance of major bacterial phyla in agricultural and natural soils from different regions is presented in **Figure 5**. The relative abundance of *Acidobacteria* was significantly greater in natural soils as compared to agricultural soils from arid (*P* < 0.001), continental (*P* < 0.0001), and temperate (*P* < 0.0001) regions. In arid regions, the relative abundance of *Acidobacteria* was nearly three times greater in natural soils as compared to agricultural soils. Similar to other regions, our meta-analysis showed higher relative abundance of *Acidobacteria* in natural vs. agricultural soils in tropical regions, however, this was not statistically significant. Our meta-analysis revealed higher relative abundance of phylum *Proteobacteria* in natural soils as compared to agriculture soils from all the studied regions. This trend was significant in soil from continental (*P* < 0.01), temperate (*P* < 0.0001), and tropical regions (*P* < 0.01). Our analysis further revealed significantly higher relative abundance of *Cyanobacteria* in natural soils vs. agricultural soils from arid (*P* < 0.0001), continental (*P* < 0.01), and temperate (*P* < 0.01) regions. Interestingly in arid regions the relative abundance of this group was approximately 6 fold higher in natural as compared to agricultural soils.

In contrast we observed higher relative abundance of *Chloroflexi* in agricultural soils as compared to natural soils across all regions. The relative abundance of this phylum was 2 and 6 fold higher in agriculture soils as compared to natural soils from arid (*P* < 0.001) and temperate regions (*P* < 0.0001), respectively.

The relative abundance of phylum *Actinobacteria* was significantly higher in natural vs. agricultural soils from continental regions while an opposite trend was observed in soils from tropical regions. Our analysis did not showed significant differences in the relative abundance of this phylum when agriculture and natural soils were compared from arid and temperate regions. *Firmicutes* showed significant differences among agricultural and natural soils only in arid regions (*P* < 0.0001) where the relative abundance was approximately 5 fold higher in agricultural soils. The members of phylum *Verrucomicrobia* were significantly more abundant in agricultural soils as compared to natural soils from continental (*P* < 0.0001) and tropical (*P* < 0.01) regions while the relative abundance of *Planctomycetes* was significantly higher in natural soils compared to agricultural soils (*P* < 0.01) from continental regions (**Figure 5**).

#### Correlations between the NPP and Soil Properties with the Relative Abundance of Different Bacterial Phyla Across Different Regions

The correlations between the NPP and soil properties with the microbial data are presented in Supplementary Table S1. Bacterial diversity of the arid regions was significantly correlated with NPP (ρ = 0.520; *P* < 0.001). However, in our analysis we did not observe the same correlation in other regions. In arid regions, the relative abundance of phylum *Chloroflexi* (ρ = -0.213; *P* = 0.037) and *Proteobacteria* (ρ = 0.283; *P* < 0.005) was correlated with NPP. The relative abundance of phylum *Cyanobacteria* was negatively correlated with total soil C (ρ = 0.206; *P* < 0.044) and the C:N ratio of arid regions.

Our analysis showed an increase in the number of correlations between the relative abundance of various bacterial phyla, NPP and soil properties in continental and temperate regions. For example, in continental regions significant correlations were observed between the NPP and the relative abundance of *Acidobacteria* (ρ = 0.347; *P* < 0.001), *Actinobacteria* (ρ = -0.190; *P* = 0.016), *Chloroflexi* (ρ = -0.276; *P* < 0.001), and *Firmicutes* (ρ = 0.336; *P* < 0.001). In temperate regions, NPP and soils properties were correlated with the relative abundance of many different bacterial phyla. For example, in temperate regions NPP was significantly correlated with the relative abundance of all bacterial phyla except for *Plantomycetes*. Soil C was correlated with the relative abundance of *Acidobacteria*, *Firmicutes*, *Verrucomicrobia*, and *Proteobacteria*. In fact, in temperate regions the relative abundance of phylum *Acidobacteria*, *Firmicutes*, and *Proteobacteria* was significantly correlated with NPP and all soil properties analyzed in this meta-analysis. In tropical regions, NPP was correlated with the abundance of *Chloroflexi* (ρ = 0.346; *P* = 0.014) and *Verrucomicrobia* (ρ = 0.294; *P* = 0.038). With respect to this region, the data showed no significant correlation between total soil C and the relative abundance of different bacterial phyla.

## Discussion

### NPP Differed between Agricultural and Natural Systems Only in Continental and Temperate Biomes

Terrestrial NPP represents the total annual growth of land vegetation and is the basic resource for food, fiber, and energy (Vitousek et al., 1986; Running, 2012; Krausmann et al., 2013). In addition, terrestrial NPP is also a major component of the global C cycle, and a critical precursor to net C storage. Changes in NPP due to agricultural conversion could result in either enhancing or mitigating increments in atmospheric CO_2_ concentrations and climate warming (Fargione et al., 2008; Searchinger et al., 2008). Latitudinal control of insolation (solar radiation that reaches earth surface) on photosynthesis results in a noticeable decrease in NPP from tropical ecosystems to those in the middle or higher latitudes (**Figure 2**). It is generally assumed that agricultural ecosystems are significantly less productive (e.g., by harvest-induced reductions in growing season length) than natural systems in the same environment (Smith et al., 2014). On the contrary it can also been argued that agricultural conversion at a local scale can increase NPP (e.g., by management inputs that reduce biophysical growth limitations) (Long et al., 2006). In our analysis we observed a significant reduction in NPP in agro-ecosystems as compared to natural ecosystems in continental and temperate environments (**Figure 2**). In similar environments, Smith et al. (2014) have reported a significant decrease in NPP due to agricultural conversion that was independent of conversion type, management intensity, crop type, or regions. Our analysis revealed a decrease in NPP in agro-ecosystems in tropical regions (**Figure 2**), however it was not as steep as reported by other workers (Smith et al., 2014). As most of our sites in tropical regions were situated in the industrialized west and Asia, the non-significant decrease in NPP in agricultural sites might be due to the relatively intensive management practices and crop types which could contribute to higher rates of productivity that more closely match those of natural vegetation (Gelfand et al., 2013; Smith et al., 2014). Similarly, in arid regions our analysis showed no differences between NPP of agricultural and natural systems (**Figure 2**). It seems that in nutrient poor systems, such as arid system climate constrains do not allow an increase in NPP. In arid regions, water availability will be the major constraint on NPP and the plants will be more sensitive to precipitation variation than soil management (Zhu and Southworth, 2013).

### Trends Obtained from Properties Linked to Soil Nutritional Health Were Not Consistent in Agriculture vs. Natural Systems among All the Climatic Regions

Agriculture practices generally results in a decline in soil nutrients. However, nutrients inputs, from both natural and synthetic sources can improve plant growth that increases organic matter returns leading to improvement in soil quality (Smith et al., 2015). Changes in soil properties can vary markedly with type of land cover, climate, and method, extent of vegetation removal (e.g., land clearing, fires, mechanical harvest), and management post harvests. Here we discuss trends obtained from our meta-analysis on the soil chemical properties of agricultural vs. natural systems in different climatic regions.

#### Soil Carbon

As the dominant land-use change during the past century, conversion of natural systems for agricultural production has greatly altered soil C dynamics at ecosystem, regional, and global scales (Foley et al., 2005; Bala et al., 2007; Don et al., 2011; Yonekura et al., 2012; Zhang et al., 2015). The depletion of soil total C due to the intensification of agriculture and land-use change from natural to croplands is exacerbated through agricultural practices with low return of organic material and other various factors including oxidation/mineralization, leaching and erosion (Post and Kwon, 2000; Wu et al., 2003; Lal, 2004; Zhang et al., 2015). In a meta-analysis, Guo and Gifford (2002) showed that the conversion of native forests and pastures to croplands reduced soil C stocks by 42 and 59%, respectively. The results varied, however, depending on factors such as annual precipitation, plant species and, the length of study periods. Our analysis indicated that total C % of agricultural soils were lower as compared to natural soils in temperate regions (**Figure 3B**). However, no significant difference in total C % in agricultural vs. natural systems were observed in other regions.

Previous studies have reported negative, positive, and negligible effects of land conversion on soil C content (Bashkin and Binkley, 1998; Vesterdal et al., 2002; Yang et al., 2011; Zhang et al., 2015). For example, 13% of the croplands included in a meta-analysis on the impact of tropical land use change on soil organic matter reported similar to higher soil C stocks in agricultural soils than forests (Don et al., 2011). The different sampling schemes, estimation methods, and the complexity of factors affecting soil C dynamics are attributed to the inconsistency in various studies (Don et al., 2011; Li et al., 2012). Following the land-use change, litter input from new vegetation will be terminated and replaced by litter from new vegetation, while the soil C derived from the former litter would be decomposed and mineralized by soil microbes (Zhang et al., 2013). Thus soil C stocks would be controlled not only by the degradation of old C (soil C previous to conversion) but also by the addition of new soil C (C derived from new vegetation after land use) (Del Galdo et al., 2003; Mendez-Millan et al., 2014). Our observations, particularly in Continental and Tropical regions, is in contrast to most previous studies (Guo and Gifford, 2002; Don et al., 2011) that have reported significant lower soil C in agricultural soil as compared to natural soils. This discrepancy may arise due to differences in management practices and disturbance regimes including tillage, residue retention, grazing and the duration of change in land use. Wiesmeier et al. (2015) has reported that soil cultivation may not generally result in the strong decline in soil C content, as management practices such as tillage probably promote the formation of organo-mineral associations and relocation of soil C with depth may decrease decomposition. No significant change in soil C in agricultural soils in arid/tropical regions results from boosted productivity and higher turnover rates adding more C to the soil due to organic manure/fertilizer application as well as the effect of crop residue, and irrigation regimes (Zhang et al., 2013).

#### Soil N

Conversion of natural lands into arable lands is not only characterized by losses of ecosystem C stocks, but also by significant losses of ecosystem N stocks along hydrological pathways, gaseous volatilization or through erosion (Tiessen et al., 1982; McLauchlan, 2006). A meta-analysis using mainly data from tropical sites indicated that the average loss of soil N after conversion of forests to croplands was 15% (Murty et al., 2002). Dalal et al. (2013) reported that conversion of native vegetation to perennial pasture and cropland in Australia resulted in N losses of more than 20 and 38%, respectively. Our analysis did not revealed significant differences in soil N % between agricultural and natural soils from continental, temperate, and tropical regions. We argue that the extensive use of chemical N fertilizer in agricultural soils will compensate for N losses through natural processes thereby maintaining total soil N concentrations to the levels similar to natural soils. In addition, the introduction of leguminous plants to crop rotations (Tiessen et al., 1982) or the application of organic fertilizers (Griffin et al., 2005), can support an increase in N stocks. Our analysis showed a significant higher total soil N in agricultural systems from arid regions compared to natural systems. In arid regions SOC and N stocks have been reported to depend strongly on soil types with strong interactions between soil type and land use (Mayes et al., 2014). Increases in the soil N in arid regions might also be the result of preference to grow leguminous crops which have a lower water requirement (Creswell and Martin, 1998).

#### Soil pH

Comparing soils from a similar climate in tropical, continental, and temperate regions, soils from agricultural systems tend to be more alkaline than natural soils. Liming in agricultural soils is also one of the major factors leading to an increase in soil pH (Armstrong et al., 2015). The greatest (positive) effects with pH were seen in the acidic soils, however, in arid regions where the pH tends to be more alkaline, our analysis showed no significant differences between agricultural vs. natural soils suggesting that impact of agricultural practices was soil dependent. Contrary to agricultural systems, natural ecosystems trend to be more acid in general. This difference in acidity can be generated through several mechanisms, including increased production of organic acids or through the generation of carbonic acid from higher rates of autotrophic respiration in natural soils (Richter and Markewitz, 1995). The increased acidity of forests may also be caused by increased uptake of cations by trees and consequent changes in the proportions of cations adsorbed to the soil exchange complex (Jobbagy and Jackson, 2004). Berthrong et al. (2009) have reported that higher acidity in natural soils can also be driven by changes in the proportions of cations such as Ca, Mg, Na, and K.

### Response of Soil Bacterial Community

It could be argued that our analyses suffer from biases such as those related to the different primer sets used by the studies included in our database. However, results from our previous study (Delgado-Baquerizo et al., 2016b) clearly demonstrate that primers pairs, sequencing platform, and the method of soil sampling does not significantly alter the microbial diversity and relative abundance of major soil bacterial phyla and that next generation sequencing data can be as useful as other available data to evaluate global patterns in microbial ecology. We argue that the point of variability in the results on the relative abundance of major bacterial phyla using different primer-set (Engelbrektson et al., 2010; Cai et al., 2013; Fredriksson et al., 2013) can be critical in local studies as in these cases the variability among primer sets may overlap the spatial variability within a particular plot or the effects of a given treatment on the abundance of these bacteria. However, small changes in relative abundance of different phyla that could be attributed to using different primer sets (Engelbrektson et al., 2010; Cai et al., 2013; Fredriksson et al., 2013) is unlikely to bias results from a global-scale meta-analysis like the one performed in the present study (Delgado-Baquerizo et al., 2016b). These variations are indeed a part of the intrinsic noise one may expect in similar meta-analyses conducted with other soil microbial variables (Fierer et al., 2009; Serna-Chavez et al., 2013).

#### Microbial Diversity in Agriculture vs. Natural Systems

Understanding the mechanisms that control the extent to which soil properties and microbial communities change following the conversion of natural to agricultural systems is of paramount importance to comprehend the consequences of land use changes for soil health and agricultural productivity (Sala et al., 2000). Management practices such as tillage and crop rotation; periodic fertilization; and pesticide application generate temporal and spatial changes in soil physical and chemical properties in agricultural systems (Carbonetto et al., 2014). The agricultural systems thus represents rapidly fluctuating environments with highly variable resource gradients and greater bio-physical and chemical heterogeneity as compared to natural systems, thereby providing a wide range of niches for microbial growth. This variability and heterogeneity can result in increased diversity in agricultural systems as compared to stable natural systems. In fact our meta-analysis revealed that microbial diversity increased significantly in agricultural systems of arid and temperate regions (**Figure 4**). The fact that diversity increased or was not markedly altered (continental and tropical regions) as a consequence of agriculture activities is not unexpected. In fact, microbial communities in natural systems may be limited by nutrient availability and therefore fertilizer addition may allow colonization by new species from the regional pools (Jangid et al., 2008; Upchurch et al., 2008; Jesus et al., 2009; Lee-Cruz et al., 2013; Crowther et al., 2014; Figuerola et al., 2015). However it has been reported that although local taxonomic and phylogenetic diversity of soil bacteria increases after conversion, communities become more similar across space (Rodrigues et al., 2013; Figuerola et al., 2015). The homogenization of microbial communities in response to human activities is driven by the loss of soil bacteria with restricted ranges (endemics) from the natural systems and results in a net loss of diversity (Rodrigues et al., 2013; Figuerola et al., 2015). As soil microbial diversity drives multiple ecosystem functions related to plant productivity (Delgado-Baquerizo et al., 2016a), we argue that microbial biodiversity loss (through homogenization of microbial community) should be taken into account when assessing the impact of land use change.

#### Relative Abundance of Major Bacterial Phyla in Agricultural vs. Natural Systems

In our analysis, despite the complex nature of soil microbial communities, we found general patterns characterizing microbial community responses to land use change at the continental scale which can provide strong framework for future experiments to generate empirical evidence. Across all regions, the relative abundance of phylum *Acidobacteria* was significantly greater in natural ecosystems as compared to agricultural systems (**Figure 5**). In contrast the relative abundance of *Verrucomicrobia* was higher in agro-ecosystems in continental, temperate and tropical regions. Interestingly both of these groups are classified as “oligotroph” (r-strategists, Fierer et al., 2007; Trivedi et al., 2013) based on lower growth rates and on a preference for growing on relatively recalcitrant forms of C. Although both *Acidobacteria* and *Verrucomicrobia* seems to be dominant groups in soil, their ecology remains poorly understood as the members of these group are difficult to culture and study in the laboratory (Bergmann et al., 2011; Fierer et al., 2013). The negative effect of agricultural systems on *Acidobacteria* may be also related to higher pH in agro-ecosystems compared to natural ecosystems.

The relative abundance of *Proteobacteria* was higher in natural soils as compared to agricultural soils in all regions apart from arid regions (**Figure 5**). Many members of *Proteobacteria* are classified as plant growth promoting bacteria that facilitates nutrient acquisition and provides protection against diseases (Lugtenberg and Kamilova, 2009). Lower relative abundance of *Proteobacteria* in agricultural soils can thus have important implications for plant productivity and soil health. Interestingly, we observed that the decrease in proportion of *Cyanobacteria* was accompanied by an increased proportion of *Chloroflexi* in agricultural systems of Arid, Continental, and Temperate regions. The metabolic flexibility of *Chloroflexi* (Strauss and Fuchs, 1993) can provide a competitive advantage against *Cyanobacteria* for limiting nutrients or physical space when they co-occur in the same environment especially in fluctuating environmental conditions in agricultural soils. Change in the ratio between *Cyanobacteria* and *Chloroflexi* has been implicated to be the result of physical disturbances that lead to the destruction of the microscale topography, decreased N availability and likely altered soil moisture retention and soil surface albedo (Kuske et al., 2012). Therefore, the relationship between these two related phyla in soil environments has to be investigated in details to develop early warning tools for soil degradation.

### Microbial Indicators of Soil Fertility and Primary Productivity

The results of our meta-analysis provide useful information about the global distribution of several groups of numerically abundant bacterial phyla in agricultural vs. natural systems across contrasting climatic regions. It was demonstrated that certain bacterial phyla responded differentially to the conversion of natural to agro-ecosystems and the trend was consistent across all studied regions. For example, the relative abundance of *Acidobacteria* was higher in natural systems while the abundance of *Chloroflexi* was higher in agricultural systems. In our analysis, the dataset is derived from the relative abundance of major bacteria groups using next generation sequencing; however, previous studies have shown a significant correlation between the relative abundance and absolute numbers of major bacterial groups using qPCR (Trivedi et al., 2015). Our findings highlight the potential of molecular tools to identify bacterial groups that may serve as potential indicators to assess the sustainability of agricultural soil management and to monitor trends in soil condition over time.

It can be argued that the selection of indicator species based solely on the frequency of occurrence does not permit conclusions about the process in which they are involved (Figuerola et al., 2012). However, as discussed above the relative abundance of groups showing consistent trends in abundance in natural and agricultural systems across all the regions can be inferred by their trophic life-strategies and related to soil physio-chemical parameters. Therefore, it can be validly postulated that the abundance of these groups reflects true habitat specialization underlying ecological selection based on soil management. The abundance of the suggested bacterial phyla is easily measured since well-established molecular and conventional culturing protocols for quantification are available (Fierer et al., 2012); they are sensitive to soil management actions and are integrative, i.e., provide adequate coverage across a relatively wide range of ecological variables, soil types, climate, crop sequence, etc. Herein we provide a regional scale framework for developing appropriate tests for simple monitoring of proposed candidate biological indicators that can be integrated into a minimum dataset, to facilitate measuring the impact of agriculture on soil health. This will allow the development of base-line values and ranges to incorporate microbial indicators in management decisions. However, significant background work including identifying context of monitoring (aridity vs. productivity), selection parameters for biological indicators (positive or negative) need to be tested and validated before an efficient indicator of primary productivity can be developed for monitoring purpose.

## Conclusion

We provide a detailed characterization of how bacterial communities change following the conversion of natural to agricultural systems, and reveal community-scale trends that hold across tropical, temperate, continental, and arid biomes. We propose that measures of microbial abundance may serve as indicators of changing to soil health before actual decline in physico-chemical properties are detected. Although our meta-analysis is derived from comprehensive datasets on the effect of agriculture on soil properties and the relative abundance of microbial taxa, this global dataset does not mirror the current hot spots of land use changes. New efforts are needed to quantify the effect of land use changes in South East Asia and Africa, also taking to account the carbon-rich wetland forests and degradation cascades within land-use classes. Nevertheless our meta-analysis provides clear signals on the predictable nature of the microbial community responses to land-use types which can be used to conceptualize future studies on understanding of human decision-making for soil health and biodiversity.

## Author Contributions

PT and MD-B designed this study and performed the meta-analysis in consultation with BS. Statistical analyses were done by PT and MD-B in consultation with BS and IA. PT wrote the article, with contributions from all co-authors.

## Conflict of Interest Statement

The authors declare that the research was conducted in the absence of any commercial or financial relationships that could be construed as a potential conflict of interest.

## Footnotes

1 http://www.scopus.com/

2 http://www.neoninc.org/

3 www.cm2bl.org

4 http://neo.sci.gsfc.nasa.gov/
