# Supplementary material for: Response of Soil Properties and Microbial Communities to Agriculture: Implications for Primary Productivity and Soil Health Indicators
Source: Front Plant Sci. 2016 Jul 12;7:990. doi: 10.3389/fpls.2016.00990 (PMC4940416; doi:10.3389/fpls.2016.00990)
Supplement: Supplementary file 1 [file Table_1.DOCX]

Supplementary Table 1. Spearman correlations between microbial components (diversity and composition) and ecosystem services for the different climates considered in this study.

| **Climate** | **Parameter** | **Ecosystem function** | **H’** | **Acidobacteria** | **Actinobacteria** | **Chloroflexi** | **Cyanobacteria** | **Firmicutes** | **Verrucomicrobia** | **Proteobacteria** | **Plantomycetes** |
| --- | --- | --- | --- | --- | --- | --- | --- | --- | --- | --- | --- |
| **Arid** | **ρ** | **Primary net productivity** | 0.542 | -0.055 | -0.050 | -0.213 | -0.181 | -0.017 | -0.166 | 0.283 | 0.035 |
|  | **P-value** |  | <0.001 | 0.596 | 0.630 | 0.037 | 0.078 | 0.871 | 0.106 | 0.005 | 0.734 |
|  | **ρ** | **pH** | -0.033 | 0.017 | 0.132 | 0.036 | -0.012 | 0.056 | 0.058 | -0.202 | 0.002 |
|  | **P-value** |  | 0.804 | 0.871 | 0.199 | 0.725 | 0.904 | 0.591 | 0.572 | 0.048 | 0.986 |
|  | **ρ** | **Soil total C** | 0.288 | 0.078 | -0.104 | -0.089 | -0.206 | -0.078 | -0.046 | -0.061 | 0.145 |
|  | **P-value** |  | 0.027 | 0.451 | 0.312 | 0.389 | 0.044 | 0.453 | 0.659 | 0.554 | 0.159 |
|  | **ρ** | **C:N ratio** | 0.087 | 0.086 | -0.137 | -0.088 | -0.272 | -0.162 | 0.082 | -0.073 | 0.230 |
|  | **P-value** |  | 0.512 | 0.403 | 0.184 | 0.394 | 0.007 | 0.114 | 0.426 | 0.482 | 0.024 |
|  | **ρ** | **Total N** | 0.289 | 0.007 | 0.008 | -0.209 | -0.105 | -0.123 | -0.247 | 0.037 | 0.147 |
|  | **P-value** |  | 0.026 | 0.944 | 0.939 | 0.041 | 0.308 | 0.233 | 0.015 | 0.722 | 0.154 |
| **Continental** | **ρ** | **Primary net productivity** | -0.101 | 0.347 | -0.190 | -0.276 | -0.098 | 0.336 | -0.049 | -0.018 | 0.004 |
|  | **P-value** |  | 0.325 | <0.001 | 0.016 | <0.001 | 0.216 | <0.001 | 0.539 | 0.821 | 0.960 |
|  | **ρ** | **pH** | 0.181 | -0.509 | -0.014 | 0.262 | 0.174 | -0.108 | 0.098 | 0.083 | -0.199 |
|  | **P-value** |  | 0.077 | <0.001 | 0.864 | 0.001 | 0.027 | 0.172 | 0.212 | 0.293 | 0.011 |
|  | **ρ** | **Soil total C** | -0.266 | 0.215 | -0.160 | 0.081 | -0.335 | -0.054 | 0.162 | -0.152 | -0.201 |
|  | **P-value** |  | 0.009 | 0.006 | 0.042 | 0.305 | <0.001 | 0.492 | 0.039 | 0.054 | 0.010 |
|  | **ρ** | **C:N ratio** | -0.204 | 0.319 | -0.140 | -0.155 | -0.228 | 0.068 | 0.091 | -0.083 | -0.161 |
|  | **P-value** |  | 0.046 | <0.001 | 0.076 | 0.049 | 0.004 | 0.391 | 0.250 | 0.296 | 0.040 |
|  | **ρ** | **Total N** | -0.286 | 0.094 | -0.089 | 0.135 | -0.299 | -0.123 | 0.124 | -0.158 | -0.133 |
|  | **P-value** |  | 0.005 | 0.232 | 0.260 | 0.088 | <0.001 | 0.118 | 0.117 | 0.044 | 0.092 |
| **Temperate** | **ρ** | **Primary net productivity** | -0.068 | 0.293 | -0.187 | -0.214 | -0.162 | -0.196 | -0.313 | 0.336 | 0.109 |
|  | **P-value** |  | 0.421 | <0.001 | 0.007 | 0.002 | 0.019 | 0.004 | <0.001 | <0.001 | 0.117 |
|  | **ρ** | **pH** | 0.212 | -0.436 | 0.061 | 0.366 | 0.120 | 0.449 | 0.106 | -0.435 | -0.421 |
|  | **P-value** |  | 0.011 | <0.001 | 0.382 | <0.001 | 0.082 | <0.001 | 0.125 | <0.001 | <0.001 |
|  | **ρ** | **Soil total C** | 0.060 | 0.237 | -0.106 | -0.057 | 0.056 | -0.355 | -0.190 | 0.270 | 0.055 |
|  | **P-value** |  | 0.473 | 0.001 | 0.126 | 0.413 | 0.417 | <0.001 | 0.006 | <0.001 | 0.425 |
|  | **ρ** | **C:N ratio** | -0.362 | 0.181 | 0.035 | -0.422 | 0.168 | -0.352 | 0.038 | 0.344 | 0.195 |
|  | **P-value** |  | <0.001 | 0.009 | 0.615 | <0.001 | 0.015 | <0.001 | 0.583 | <0.001 | 0.005 |
|  | **ρ** | **Total N** | 0.186 | 0.189 | -0.144 | 0.088 | 0.019 | -0.249 | -0.218 | 0.234 | -0.013 |
|  | **P-value** |  | 0.026 | 0.006 | 0.037 | 0.204 | 0.782 | <0.001 | 0.001 | 0.001 | 0.854 |
| **Tropical** | **ρ** | **Primary net productivity** | 0.160 | 0.083 | -0.225 | 0.346 | 0.171 | 0.037 | 0.294 | -0.160 | 0.149 |
|  | **P-value** |  | 0.389 | 0.565 | 0.116 | 0.014 | 0.236 | 0.799 | 0.038 | 0.268 | 0.300 |
|  | **ρ** | **pH** | 0.186 | -0.550 | 0.205 | -0.134 | -0.043 | 0.384 | -0.239 | 0.196 | -0.252 |
|  | **P-value** |  | 0.316 | <0.001 | 0.153 | 0.355 | 0.765 | 0.006 | 0.095 | 0.172 | 0.078 |
|  | **ρ** | **Soil total C** | 0.036 | 0.110 | 0.126 | -0.168 | 0.236 | 0.199 | 0.011 | 0.107 | -0.293 |
|  | **P-value** |  | 0.849 | 0.446 | 0.383 | 0.245 | 0.099 | 0.166 | 0.939 | 0.459 | 0.039 |
|  | **ρ** | **C:N ratio** | -0.090 | 0.102 | 0.059 | -0.045 | 0.369 | 0.225 | 0.285 | -0.146 | -0.094 |
|  | **P-value** |  | 0.630 | 0.483 | 0.684 | 0.758 | 0.008 | 0.115 | 0.044 | 0.311 | 0.516 |
|  | **p** | **Total N** | 0.187 | 0.038 | 0.057 | -0.245 | -0.085 | 0.045 | -0.341 | 0.318 | -0.366 |
|  | **P-value** |  | 0.315 | 0.794 | 0.696 | 0.086 | 0.558 | 0.756 | 0.015 | 0.024 | 0.009 |
